# Mass Media and the Contagion of Fear: The Case of Ebola in America

**DOI:** 10.1371/journal.pone.0129179

**Published:** 2015-06-11

**Authors:** Sherry Towers, Shehzad Afzal, Gilbert Bernal, Nadya Bliss, Shala Brown, Baltazar Espinoza, Jasmine Jackson, Julia Judson-Garcia, Maryam Khan, Michael Lin, Robert Mamada, Victor M. Moreno, Fereshteh Nazari, Kamaldeen Okuneye, Mary L. Ross, Claudia Rodriguez, Jan Medlock, David Ebert, Carlos Castillo-Chavez

**Affiliations:** 1 Arizona State University, Tempe, AZ, U. S. A.; 2 Purdue University, West Lafayette, IN, U. S. A.; 3 Oregon State University, Corvallis, OR, U. S. A.; Hellas, GREECE

## Abstract

**Background:**

In the weeks following the first imported case of Ebola in the U. S. on September 29, 2014, coverage of the very limited outbreak dominated the news media, in a manner quite disproportionate to the actual threat to national public health; by the end of October, 2014, there were only four laboratory confirmed cases of Ebola in the entire nation. Public interest in these events was high, as reflected in the millions of Ebola-related Internet searches and tweets performed in the month following the first confirmed case. Use of trending Internet searches and tweets has been proposed in the past for real-time prediction of outbreaks (a field referred to as “digital epidemiology”), but accounting for the biases of public panic has been problematic. In the case of the limited U. S. Ebola outbreak, we know that the Ebola-related searches and tweets originating the U. S. during the outbreak were due *only* to public interest or panic, providing an unprecedented means to determine how these dynamics affect such data, and how news media may be driving these trends.

**Methodology:**

We examine daily Ebola-related Internet search and Twitter data in the U. S. during the six week period ending Oct 31, 2014. TV news coverage data were obtained from the daily number of Ebola-related news videos appearing on two major news networks. We fit the parameters of a mathematical contagion model to the data to determine if the news coverage was a significant factor in the temporal patterns in Ebola-related Internet and Twitter data.

**Conclusions:**

We find significant evidence of contagion, with each Ebola-related news video inspiring tens of thousands of Ebola-related tweets and Internet searches. Between 65% to 76% of the variance in all samples is described by the news media contagion model.

## Introduction

Use of Google search trend data to predict disease outbreaks was first proposed in 2006 [1], and subsequently developed further in the years prior to the 2009 A/H1N1 outbreak [2, 3]. This resulted in the advent of the Google Flu Trends online application, which uses a combination of flu-related Internet search activity to predict, in real-time, flu outbreaks in the U. S. and other countries worldwide (see http://www.google.org/flutrends/, accessed February 9, 2015).

Twitter is a social networking and micro-blogging service that enables its millions of users to send and read each other’s tweets, consisting of short, 140-character messages. The service currently has over 280 million monthly active users, sending on average around 500 million tweets per day (see http://about.twitter.com/company, accessed February 9, 2015). Twitter was first proposed as a means to track disease in real-time in a 2011 publication that looked retrospectively at flu-related tweets during the 2009 A/H1N1 pandemic [4].

Since these initial analyses, the field of “digital epidemiology” has rapidly expanded [5–8]. For example, Twitter data has been used to examine cholera outbreaks [9], vaccination sentiments [10], and proposed for use for global infectious disease surveillance [11]. Google search data has been used for real-time forecasting of Dengue outbreaks [12, 13], Listeria outbreaks [14], the spread of tuberculosis [15], consumer behavior [16], and the economy [17].

However, there have been some failings of these methods, leading to some concern about their applicability in an outbreak of an emerging infectious disease. At the beginning of the 2009 A/H1N1 outbreak, Google Flu Trends was not accurate in predicting the progression of the pandemic, due to information seeking behavior that was likely induced by mass media coverage [18]. Indeed, Chew et al (2009) found that some peaks in flu-related Twitter activity during the 2009 A/H1N1 pandemic were correlated to the timing of major news stories about the pandemic [19]. Additionally, during the severe 2012–13 influenza season, Google Flu Trends did not accurately predict the course of the outbreak, seriously overestimating the disease burden in the population. It has been suggested that the problems may have been due to widespread media coverage, including the declaration of a public-health emergency by New York state [20]. It has also been noted that news media is effective in “pulsing” of public opinion during election times [21], as expressed on social media.

Despite the fact that evidence of the influence of news media on Twitter and Internet search patterns has been repeatedly observed, up until now it has been extraordinarily difficult to correct for this effect because the patterns are also intertwined with temporal patterns due to the other dynamics of interest, such as the actual spread of a disease within a population.

**Fig 1:**
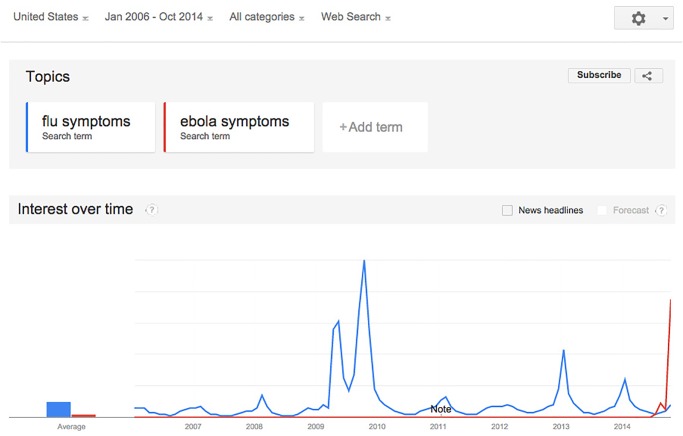
Comparison of the 2014 Ebola-related Google search trends to influenza-related search trends during the 2009 A/H1N1 pandemic. The relative interest in Ebola-related searches during the month of October 2014 rivaled the flu-related searches at the beginning of the A/H1N1 pandemic.

During the recent very limited U. S. Ebola outbreak, the popularity of Ebola-related Google searches in the U. S. rivaled that of flu-related searches during the 2009 A/H1N1 pandemic (see Fig 1), but we can be confident that none (or virtually none) of the Ebola-related U. S. Internet searches or tweets arose from actual victims of Ebola in the U. S. The situation thus provides an excellent means to determine how public interest, curiosity, or panic regarding a certain topic affects social media and Internet search dynamics, and allows us to examine the influence of news media on these trends. The results of this study will thus help to inform future digital epidemiological analyses of outbreak data, possibly allowing for the correction of the effects of benign interest, information-seeking behavior, or public hysteria.

In this analysis we employ a mathematical model of contagion to simulate the potential influence of Ebola-related news videos on peoples’ tendency to perform Ebola-related Internet searches or tweets. The advantage of such a model over the use of a simple statistical regression model to explore these relationships is that a mathematical model can incorporate non-linear dynamics related to people becoming immune to news media induced interest in a topic (i.e. the model incorporates the effect of eventual boredom with a topic, no matter how many news videos are subsequently aired). For comparison purposes, however, we also present the results of statistical linear regression models examining these relationships.

As a cross-check to our analysis, we verify that public interest in Ebola (as reflected by Internet searches and tweets) does not appear to induce news media stories on the topic. We also show that peaks and valleys in the temporal trends in news media coverage tend to precede the same features in the Internet and Twitter data, not the other way around.

In the following sections we describe the sources of data used in this analysis, and describe the contagion model employed, followed by a presentation of results and discussion.

## Materials and Methods

### Data

#### Google search data

Daily trends in Ebola-related Google searches were obtained from Google Trends application program interface (API) (http://www.google.com/trends). The search terms examined were:

“Ebola”
“Ebola symptoms”
“Do I have Ebola”

These search terms were chosen to reflect varying degrees of interest in the topic, from casual curiosity to possible panic regarding the potential of personal infection with Ebola. Only searches originating in the U. S. were considered for this analysis.

The data provided by Google Trends are normalized to the total search volume on Google, and thus the data are relative and not absolute. To estimate the total number of searches, we obtained the total number of searches per month originating in the U. S. for each search term from the Google Adwords subscription service, via the Keyword Planner tool, and scaled the Google Trend data accordingly.

**Fig 2:**
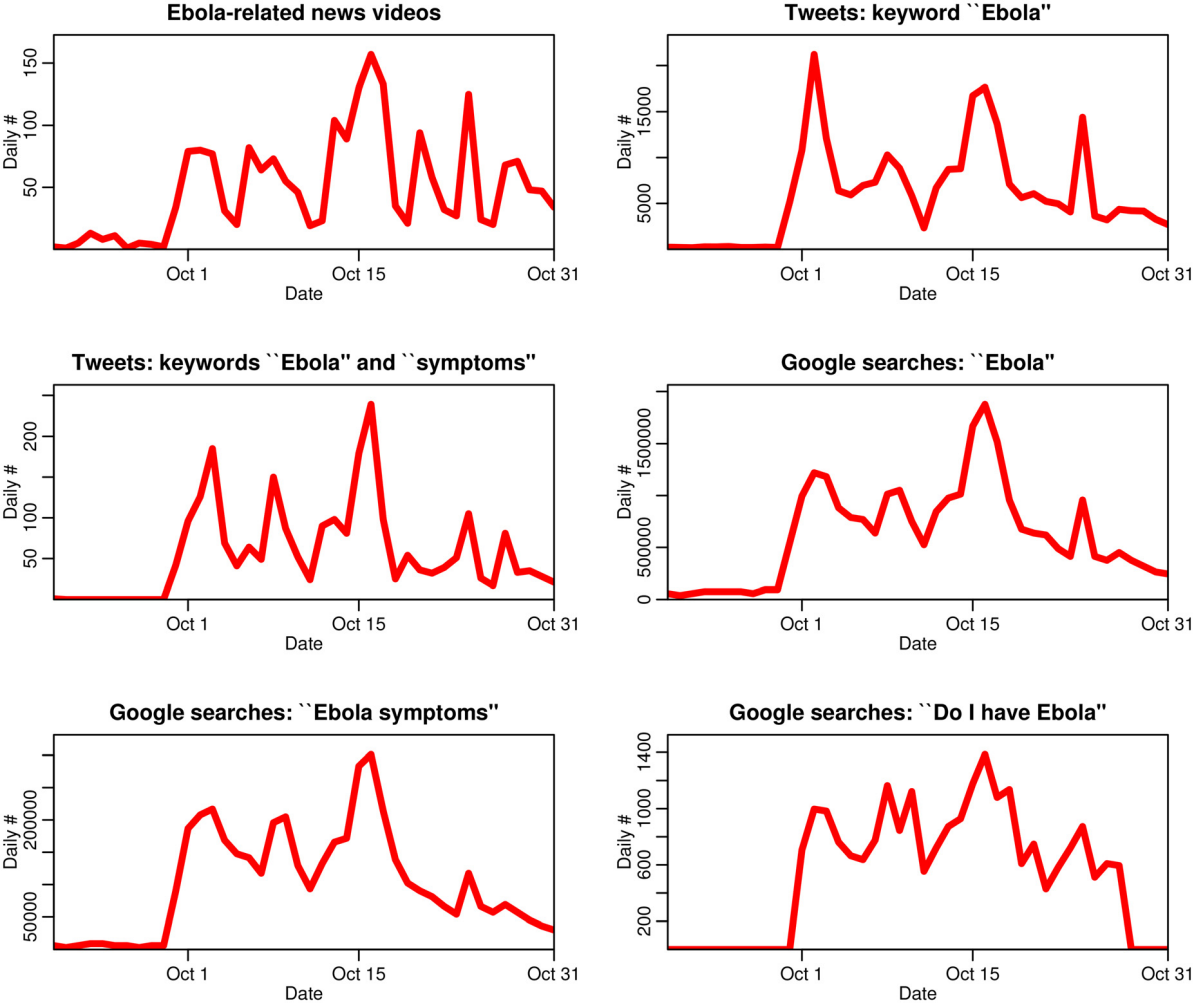
Time series of Ebola-related news media, Twitter, and Google search data used in this study. The samples consist of six weeks of data ending October 31^st^, 2014. The first case of Ebola confirmed in the U. S. occurred on September 29, 2014. The temporal trends in the data samples are highly inter-correlated, with a minimum of 70% correlation between samples.

The data are shown in Fig 2.

#### Twitter data

Daily Twitter data were obtained from a repository continuously collected by a server maintained by the Purdue center for Visual Analytics for Command, Control, and Interoperability Environments (VACCINE), which uses the Twitter streaming Application Programming Interface (API) to access up to 1% of the global stream of tweets.

From this stream, we selected tweets during the six week period ending October 31, 2014 containing the keyword “Ebola”, or the keywords “Ebola” and “symptoms”. Only English language tweets geo-located in the United States were considered, and retweets were removed from the sample, resulting in a total of 260947 tweets.

Potential bias in the temporal trends due to tweets coming from news agencies, rather than regular (aka “grassroots”) Twitter users needs to be minimized. We thus separated the tweets coming from obvious news agencies, public entities, or reporters; CNN, NBC, ABC, CBS, FOX, MSNBC, New York Times, Washington Post, Reuters, Bloomberg News, New Day, U.N. Spokesperson, and Geraldo Rivera. We also excluded tweets coming from users with usernames that included the words “TV”, “media”, “trend”, “CP” (which often stands for “City Press”), and “report” (all selections were applied such that they were not sensitive to the case of the characters in the username). This yielded a sample of 10224 tweets.

As noted in Reference [22], the Twitter accounts of news agencies compared to the accounts of grassroots users tend to have a much smaller indegree to outdegree ratio (i.e. they usually follow far fewer accounts than they have followers). Reference [22] found that this ratio was on average 0.5 for news agencies (with median 0.001), and between 1.1 to 2.6 for non-media Twitter users (with median between 0.8 to 1.6). For our particular sample, we find this ratio is 0.34 for news agency users (with median 0.007), and 1.3 for the remaining users (with median 0.95). Thus, based on both the user name, and the properties of the user account, the remaining 250723 tweets are consistent with having not come from news agencies. These remaining data are shown in Fig 2.

#### News video data

According to a 2013 Gallup poll of over 2,000 people, 55% of Americans turn to television to obtain their news [23], with the next most popular medium being the Internet (22%). According to another study done in 2014 by the Pew Research center [24], CNN, MSNBC, and Fox News are the three primary cable news networks, with Fox News and MSNBC having an 80% share of that market.

While CNN makes available online a repository of its very recent videos, they are not reliably identifiable by date. However, both Fox News and MSNBC provide tools on their websites to obtain videos related to specific topics within specific date ranges. These videos are aired both on television, and also available for online viewing. We use these data as a proxy for the temporal trends in the total amount of news media coverage related to Ebola.

From these repositories, we downloaded the daily number of Ebola-related videos for the six week period ending October 31st, 2014. The data are shown in Fig 2.

### Granger Causality

The Granger causality test is a hypothesis test for determining whether or not one time series is able to forecast another [25]. Granger causality is defined on the principle that a cause necessarily must happen prior to its effect, and that the cause contributes unique information regarding the future value of its effect.

A time series *X* is said to Granger-cause *Y* if it can be shown that *X* provides statistically significant information about the future values of *Y*. This is achieved through *F*-tests based on linear regression of *Y* on lagged values of *X* (including also lagged values of *Y*). Here, we examine the potential Granger causality of temporal patterns in the daily number of news videos on temporal patterns in Ebola-related tweets and Internet searches the following day.

Significant Granger-causality provides evidence that *X* may in fact cause *Y* (although the caveat that correlation does not equal causation applies). Note, however, that in the case that *X* does indeed cause *Y*, the time lag between cause and effect must not be too small relative to the time step of the time series in order for the effect to be detectable with the Granger-causality test.

### Contagion Model

To assess the potential contagion of Ebola-related news media in inspiring Ebola-related Google searches or tweets, we employ a Susceptible, Infected, Recovered, Susceptible, and Vector (SIRSV) compartmental model. News videos, V, can “infect” susceptible individuals with interest in the topic, that is subsequently evidenced by Internet searches or tweets. After an average period of 1/*γ* days, the individual performs an Internet search or tweet related to the topic, and then “recovers”, subsequently becoming less interested with the topic and no longer feeling a need to perform another Internet search or tweet. The model includes the potential that recovered individuals can flow back into the susceptible compartment (i.e. after a period of time the individual may once again become susceptible to being infected with interest in the topic).

The equations of the model are

$$\begin{array}{ccc} \frac{dS}{dt} & = & -\beta VS/N-\mu SI/N+\alpha R\\ \frac{dI}{dt} & = & +\beta VS/N+\mu SI/N-\gamma I\\ \frac{dR}{dt} & = & +\gamma I-\alpha R, \end{array}$$ (1)

where *N* = *S* + *I* + *R* is the U. S. population, and *V*(*t*) is the temporal evolution of the number of news videos per day. The parameter *β* is the number of Internet searches or tweets per unit time inspired by one news video shown to a completely susceptible population. The parameter *μ* represents the rate of searches inspired by things other than news media, such as people talking among themselves about a particular subject. The parameter *α* represents the rate at which recovered individuals flow back in to the susceptible compartment.

Our model is somewhat similar to the model used in Reference [26] to simulate the spread of ideas within a population, with the difference that we include an exogenous infection component, *V*, rather than assuming that contagion is only due to intrinsic infectious process within the closed population.

The system of equations in Eq 1 is numerically solved to obtain the estimated number Internet searches or tweets made per unit time, *γI*(*t*). The number of news videos varies quite dramatically by day, making numerical solutions unreliable due to stiffness of the model equations if the raw data for the daily number of news videos, *V*(*t*), are used; we thus first fit a cubic spline to the daily news video data in order to smoothly interpolate the data in finer time steps.

To estimate the model parameters that optimally describe each data sample, we use the Monte Carlo method for solution of inverse problems to randomly sample the parameters of the model from broad uniform distributions, and calculate the Pearson *χ* ^2^ goodness-of-fit statistic comparing the model to the data sample [27, 28]. The uniform distribution sampling range is chosen to be large enough to ultimately include the parameter optimal value and at least a ±5 standard deviation range about that value. In order to determine the parameter hypotheses that minimize the Pearson *χ* ^2^ goodness-of-fit statistic, this procedure is repeated at least one million times for each sample with the use of National Science Foundation Extreme Science and Engineering Discovery Environment (NSF XSEDE) high-throughput computing resources (see www.xsede.org, accessed February 9, 2015). To determine the parameter 95% confidence intervals, the Pearson *χ* ^2^ statistic is corrected for over-dispersion using the ansatz of McCullagh and Nelder (1989) [29].

If the parameter optimization procedure determines that *μ* and *α* are statistically consistent with zero (i.e. that there is no evidence of contagion within the population due to dynamics other than exposure to news videos, and immunity upon recovery is permanent), a more appropriate model is

$$\begin{array}{ccc} \frac{dS}{dt} & = & -\beta VS/N\\ \frac{dI}{dt} & = & +\beta VS/N-\gamma I\\ \frac{dR}{dt} & = & +\gamma I. \end{array}$$ (2)

In addition, if the parameter optimization procedure determines that *μ*, *α*, and 1/*γ* are statistically consistent with zero, the most appropriate model is

$$\begin{array}{ccc} \frac{dS}{dt} & = & -\beta VS/N\\ \frac{dR}{dt} & = & +\beta VS/N, \end{array}$$ (3)

where the move to the recovered class happens essentially immediately after exposure to a news video. Given that our data are aggregated by day, “immediately” in this case essentially implies a time frame smaller than a day.

Note that initially the entire population is not typically susceptible to ideation to do an Ebola-related Internet search or tweet due to exposure to an Ebola-related news story aired by Fox News or MSNBC; the people considered “susceptible” in this analysis are people who watch those networks, have access to a computer, and are also susceptible to various levels of ideated panic, ranging from simple interest or curiosity, to actually wondering if they themselves might have Ebola. In the parameter estimation procedure, we thus also estimate the initial fraction of susceptible people in the population for each kind of Ebola-related tweet or Internet search.

As a validation cross-check of the model, we optimize the model parameters to the first half of the time series, and use the resulting model to predict the temporal patterns of the remaining half. We also perform this model validation procedure for a simple linear regression fit, where the time series of the number of Internet searches and tweets are regressed on *V*. Unlike the mathematical model, the linear regression model does not include the effect of eventual boredom with a topic.

As an additional cross-check of the analysis, we swap *I* and *V* in the model, and examine the potential that Ebola-related Internet searches or tweets inspire the temporal patterns in news media coverage, rather than the other way around.

## Results and Discussion

For all data samples considered in this analysis, we find that the contagion model parameter *μ* is statistically consistent with zero (i.e. we find that there is no statistically significant evidence of contagion due to effects other than news videos). We also find that the parameter *α* is statistically consistent with zero for all samples (i.e. there is no statistically significant evidence that people return to the susceptible class after recovery). In addition, for the Twitter data samples, 1/*γ* is statistically consistent with zero, indicating that after viewing a news video the movement to the recovered compartment after doing an Ebola-related tweet occurs within a day. For these data samples, we thus fit the reduced contagion model of Eq 3.

In Table 1, we show the best-fit parameters of the contagion model fit to the data samples. In Table 2, we show the percentage of the variance, *R* ^2^, in the data samples described by the best-fit contagion model. We also show the results of the model validation procedure, showing the *R* ^2^ of the model fit to the first half of each data sample, and the *R* ^2^ of the subsequent model prediction extrapolated to the last half of the sample. The *R* ^2^ of a simple statistical model, linearly regressing the data samples on the daily number of Ebola-related news videos, is also shown. In all cases, the contagion model has better predictive power than the linear regression model.

**Table 1:** Parameters of the Ebola-related news media contagion model of Eq 2 or Eq 3 (as appropriate to the sample), fit to the Ebola-related Google searches and tweets.

	N	*f*	β*f	1/*γ* (days)
Tweets: keyword “Ebola”	251,000	0.0012 [0.0011, 0.0032]	180 [140, 210]	-
Tweets: keywords “Ebola” and “symptoms”	2,350	1*e*−05 [9.7*e*−06,2.8*e*−05]	1.7 [1.3,2.2]	-
Google searches: “Ebola”	26,100,000	0.12 [0.097,0.18]	22000 [17000, 31000]	0.7 [0.3,3.1]
Google searches: “Ebola symptoms”	4,240,000	0.017 [0.015,0.022]	4000 [3200, 5100]	0.7 [0.3,1.9]
Google searches: “Do I have Ebola”	22,200	8.8*e*−05 [7.6*e*−05,0.00014]	25 [16, 71]	3.8 [1.4,14.3]

The parameter *f* is the initial fraction of the population susceptible to news media induced Ebola interest or panic (as manifested by the particular Ebola-related Internet searches or tweets in our samples). The parameter *β* is the transmission rate, and 1/*γ* is the average time, in days between an individual viewing an Ebola-related news video, and performing an Ebola-related Google search or tweet. The average number of particular Internet searches or tweets in our samples inspired by a single news video in the initial susceptible population is *fβ*. The numbers in the square brackets represent the 95% confidence intervals.

**Table 2:** The percentage of the variance, *R* ^2^, of the Ebola-related Twitter and Google search samples described by the contagion model of Eq 2 or Eq 3 (as appropriate to the sample); shown are the *R* ^2^ of the model fit to the full sample, the first half of the sample (model validation training sample), and the extrapolated model prediction for the remaining half of the sample (model validation test sample).

	Contagion Model			Regression Model		
	*R* ^2^ full	*R* ^2^ train	*R* ^2^ test	*R* ^2^ full	*R* ^2^ train	*R* ^2^ test
Tweets: keyword “Ebola”	0.75	0.84	0.63	0.72	0.78	0.44
Tweets: keywords “Ebola” and “symptoms”	0.65	0.81	0.51	0.66	0.77	0.47
Google searches: “Ebola”	0.73	0.79	0.66	0.70	0.76	0.62
Google searches: “Ebola symptoms”	0.76	0.81	0.74	0.62	0.75	0.50
Google searches: “Do I have Ebola”	0.75	0.89	0.37	0.49	0.67	0.15

Also shown are the *R* ^2^ for the statistical model, which linearly regresses the data samples on the daily number of Ebola-related news videos.

As a cross-check, we also use the contagion model to examine the possibility that the Ebola-related Internet searches or tweets inspire news videos on the topic. The results are summarized in Table 3. In all cases, we find that the *R* ^2^ is much worse for this model compared to the model where we assume that the news videos inspire the Internet searches and tweets.

**Table 3:** The percentage of the variance, *R* ^2^, of the data samples described by the contagion model of Eq 1, assuming that the news videos, *V*, cause the patterns seen in the data (*V* → *I*).

	Contagion Model		Granger Causality test	
	*R* ^2^ *V* → *I*	*R* ^2^ *I* → *V*	p-value *V* → *I*	p-value *I* → *V*
Tweets: keyword “Ebola”	0.75	0.38	0.11	0.89
Tweets: keywords “Ebola” and “symptoms”	0.65	0.41	0.02	0.93
Google searches: “Ebola”	0.73	0.35	0.02	0.37
Google searches: “Ebola symptoms”	0.76	0.34	0.02	0.31
Google searches: “Do I have Ebola”	0.75	0.32	0.09	0.65

Also shown are the *R* ^2^ under the assumption that the temporal patterns in the data samples cause the temporal patterns in the news videos (*I* → *V*). The p-values testing for Granger causality between the various time series are also shown.

Also shown in Table 3 are the p-values of the Granger causality test that tests the hypothesis that temporal patterns in news videos Granger-cause temporal patterns in the Internet searches and tweets on the next day, and vice versa. In all cases, there is no statistically significant evidence that Ebola-related Internet searches and tweets Granger-cause temporal patterns in Ebola-related news videos, but there is evidence in several cases that the reverse is true.

**Fig 3:**
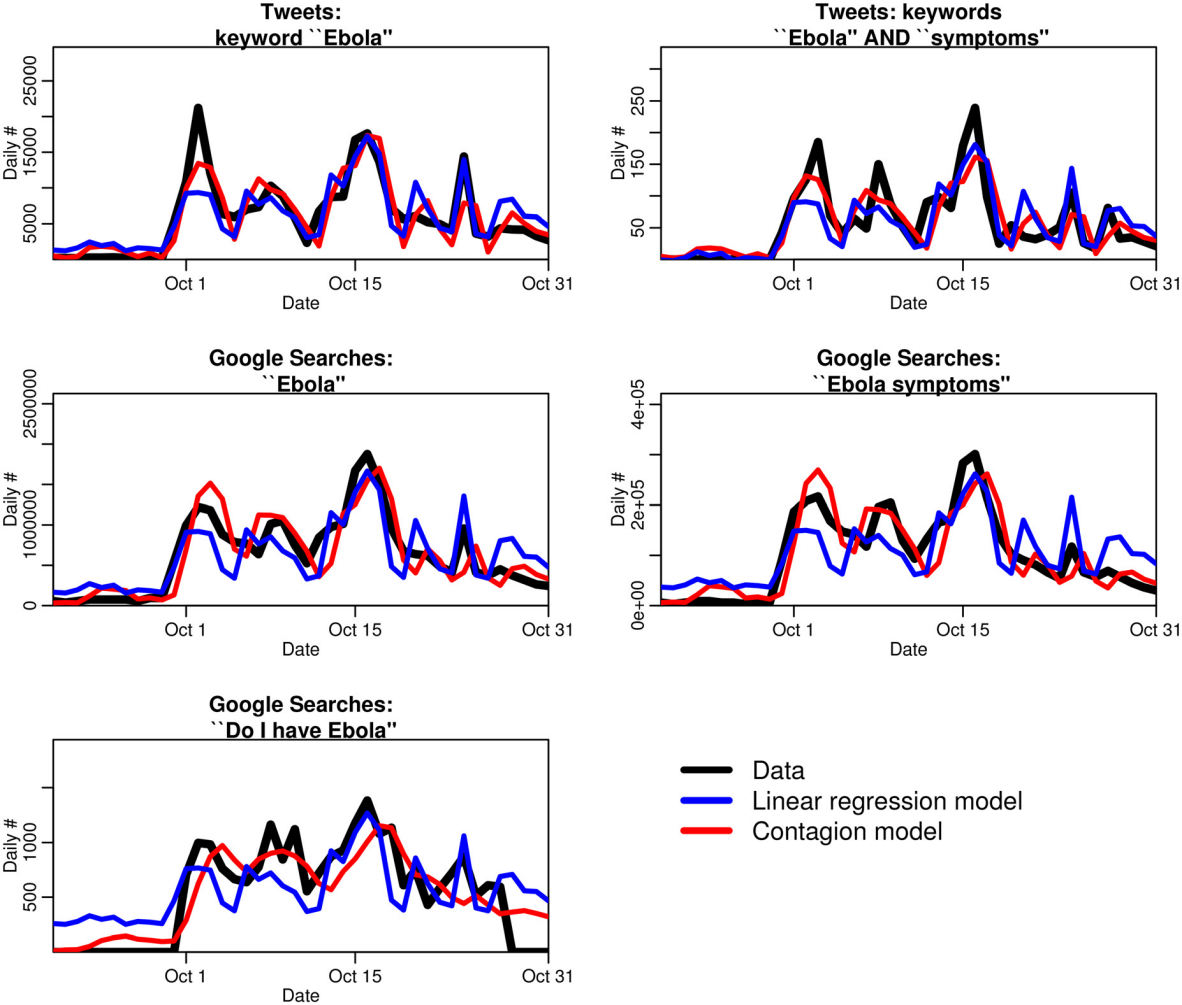
Fits of the news media contagion model, and a simple linear regression model, to the sources of data used in this study. The fits of the linear regression model (shown in blue) tend to be generally too low in the beginning and too high at the end. In contrast, the contagion model (red line) accounts for the boredom effect, where people become more and more disinclined to perform Ebola-related searches or tweets after an extended period of exposure to Ebola-related news-coverage. Incorporation of this dynamic in the model yields significantly better fits to the data compared to the regression model.

Fig 3 shows the best-fit contagion and linear regression models overlaid on the data.

## Summary

As shown in Table 2, we find that a large fraction of the variance in the data is described by the contagion model (*R* ^2^ at least 65% in all cases), and the model validation procedure shows good predictive capability for the extrapolated model to the test samples. The performance of a simple linear regression model is not as good; for all but one sample, the linear regression model yields a lower *R* ^2^ than the contagion model, and the model validation procedure reveals that the regression model has generally much poorer predictive capabilities compared to the contagion model. It is thus apparent that in modeling the dynamics of ideation due to exposure to news media, “recovery” and ultimate immunity to further ideation should be taken into account.

As shown in the best-fit model parameters Table 1, we find that a relatively large percentage of the population (up to 18%) is susceptible to ideation with what likely is simple curiosity prompting a Google search for “Ebola”, and we find that one Ebola-related news video aired by Fox News or MSNBC on average inspires tens of thousands of such searches in the American population. In contrast, a much smaller percentage of the population appears to be susceptible to ideation to perform more specific searches indicating personal concern about actually having the disease, such as Google searches for “do I have Ebola”.

The contagion model we employ assumes that people recover once performing an Ebola-related Internet search or tweeting Ebola-related information, and do not feel the need to seek or disseminate information again. Through comparison of the fit of a contagion model to the fit of a simple linear regression model that does not include the dynamics of the recovery process, we find evidence that indeed recovery and immunity to further ideation does play a role in the overall dynamics of peoples’ information seeking behavior. This is concordance with the conclusions of a previous study which examined the information seeking behavior of people during the 2009 A/H1N1 pandemic [30], and determined that information seeking became less common as the pandemic progressed.

Our model assumes that people perform just a single tweet or Internet search before moving to the “recovered” class with permanent immunity. In reality, this simplifying assumption may be violated in some cases, with some people performing several related Internet searches or tweets in a short time period. For instance, the 250,723 tweets related to Ebola used in this analysis were produced by 118,705 unique Twitter users. However, the average time between the first tweet and becoming disinterested with the topic and never tweeting again was 3 days.

In our analysis, we did not examine the sentiment expressed in the Twitter data. Previous studies have shown that negative sentiments tend to be more infectious on social networks than positive sentiments [31]; while we found no evidence of contagion of Ebola-related sentiments within the Twitter community itself in this particular analysis, it may be that sentiment, in addition to temporal patterns, may be used in a real outbreak situation to disentangle the effect of news media contagion, contagion within the social media platform, and effects due to the spread of the disease.

The vast majority of published digital epidemiology results show a positive correlation between digital data and the temporal evolution of epidemics or outbreaks; but what typically are not seen are the analyses that show no significant correlation, likely due to the “file drawer” effect where uninteresting or null results simply are not published [32, 33]. Indeed, several of the authors (ST, DE, SA) can attest to the fact that use of Twitter data to predict outbreaks is fraught with difficulties (unpublished data), and accounting for potential sources of bias is extraordinarily difficult. However, in this new age of readily accessible, and rapidly evolving, temporal and geo-spatial information in social media, digital epidemiology has a hopeful future as a tool to detect newly emerging infectious diseases, and track the spread of established diseases. The methodology is being continually refined to reduce both Type I and Type II errors (i.e. false positives and false negatives), and efforts are being made to understand the potential biases of the methods. With our analysis, we have explored a source of major potential bias when applying digital epidemiological methodology to emerging disease outbreaks; while media-induced panic is certainly not the only source of bias in such situations, we hope the results of our study will be informative for future analyses.

## Supporting Information

S1 Data The file S1_Data.csv is a file containing the data used in this study. (CSV)
